# A closed-loop spinal cord stimulation system in a patient with an implantable cardioverter defibrillator

**DOI:** 10.1097/PR9.0000000000001302

**Published:** 2025-06-06

**Authors:** Estefanía Romero-Serrano, Carlos Delgado Navarro, Blanca Quesada Ocete, Jose De Andrés

**Affiliations:** a Department of Anesthesiology and Pain Medicine, Hospital de Manises, Valencia, Spain; b Department of Anesthesiology, Critical Care and Pain Management, Hospital General Universitario de Valencia, Valencia, Spain; c Department of Cardiology, Hospital General Universitario de Valencia, Valencia, Spain

**Keywords:** Spinal cord stimulation, Implantable cardioverter defibrillator, Pacemaker, Chronic pain, Interference

## Abstract

**Introduction:**

Spinal cord stimulation (SCS) devices and implantable cardioverter defibrillators (ICDs) are well-established therapies for chronic pain management and mortality prevention in cardiovascular pathologies, respectively. As both devices rely on electrical current, there are concerns regarding potential interference between them that could lead to severe consequences.

**Methods:**

In this case report, we describe the case of a 50-year-old woman who had been implanted with an ICD for 5 years due to ischemic cardiopathy and class III heart failure symptoms, and who was referred to the Pain Unit after 4 years of persistent pain on the soles of her feet, which had not responded to opioids nor to percutaneous procedures. Thus, after being deemed suitable by anesthesiologists and obtaining psychological approval, she underwent implantation of an SCS closed-loop system.

**Results:**

Three months after the procedure, she reported an 80% relief of her pain. Both devices effectively functioned at all times, and no interference was observed.

**Conclusion:**

Pain management using a closed-loop SCS in a patient with an ICD can be safely performed without interference between the devices.

## Abstract 2

Pain management using a closed-loop spinal cord stimulation in a patient with an implantable cardioverter defibrillator can be safely performed without interference between the devices.

## 1. Introduction

Spinal cord stimulation (SCS) is a well-established therapy for managing chronic pain caused by a growing number of underlying pathologies. Technologies have been refined and further advanced, with closed-loop systems gaining popularity in recent years. By using evoked compound action potential (ECAP)—the patient's neural response to electrical stimulation—as part of a feedback mechanism, closed-loop systems ensure consistent spinal cord activation. They continuously regulate the stimulation current (with an average of 3.5 million adjustments per day) to maintain a target ECAP amplitude during physiological changes and movement. This innovation eliminates the need for patients to manually adjust stimulation parameters while performing daily activities.^4,5^

On the other hand, implantable cardioverter defibrillators (ICDs) have been shown to improve mortality rates in patients with ischemic heart disease compared with medical treatment alone. Thus, their use is on the rise: in Spain, 168 ICDs were implanted per 1 million inhabitants in 2022, representing a 2.6% increase compared with 2021, while in Europe, up to 296 ICDs per 1 million inhabitants were implanted in 2022.^8,10^

Given the growing demand for both therapies, it is crucial to analyze the clinical scenario of patients who benefit from both devices simultaneously. Potential interference between these devices could result in inadequate pain management or damage to the SCS, ultimately necessitating device replacement and additional surgical procedures, with the associated personal impact on the patient and economic burden on the hospital. More concerningly, interference from an SCS could compromise cardiac devices, such as pacemakers or ICDs, by impairing their ability to pace or causing inappropriate shock delivery. Although several case reports on the combined use of ICDs and SCS have been published—most of which reported no significant interference—there remains a notable scarcity of large-sample studies.^1,5^ Furthermore, to the best of the authors' knowledge, this is the first published case reporting the implantation of a closed-loop SCS system in a patient with an ICD.

## 2. Clinical case

A 50-year-old woman was referred to the multidisciplinary Pain Unit of our hospital due to persistent neuropathic pain in the soles of the feet, which had been present for 4 years. She had a history of ischemic heart disease and class III heart failure symptoms, requiring the implantation of a monocameral ICD Volta 1CR65 (MicroPort, Shanghai, China) without pacing function. Her neuropathic pain began in the intensive care unit during her recovery from this procedure. Over the 4 years of pain, she was treated with gabapentin (600 mg/d), capsaicin cream (0.75 mg/g), 4 applications of capsaicin patches (179 mg), and tapentadol (progressively increased from 50 mg/d to 150 mg/d), all of which provided only temporary partial relief. She also underwent percutaneous interventions, including radiofrequency denervation of the sciatic popliteal nerve and an S1 transforaminal epidural injection, with similarly limited results. After an evaluation by Pain Unit specialists using SCS prediction tools and psychological assessment, she was deemed suitable for the implantation of an SCS system. Specifically, she received the Evoke System (Saluda Medical, Artarmon, Australia), a closed-loop device based on ECAP technology.

**Figure 1.**
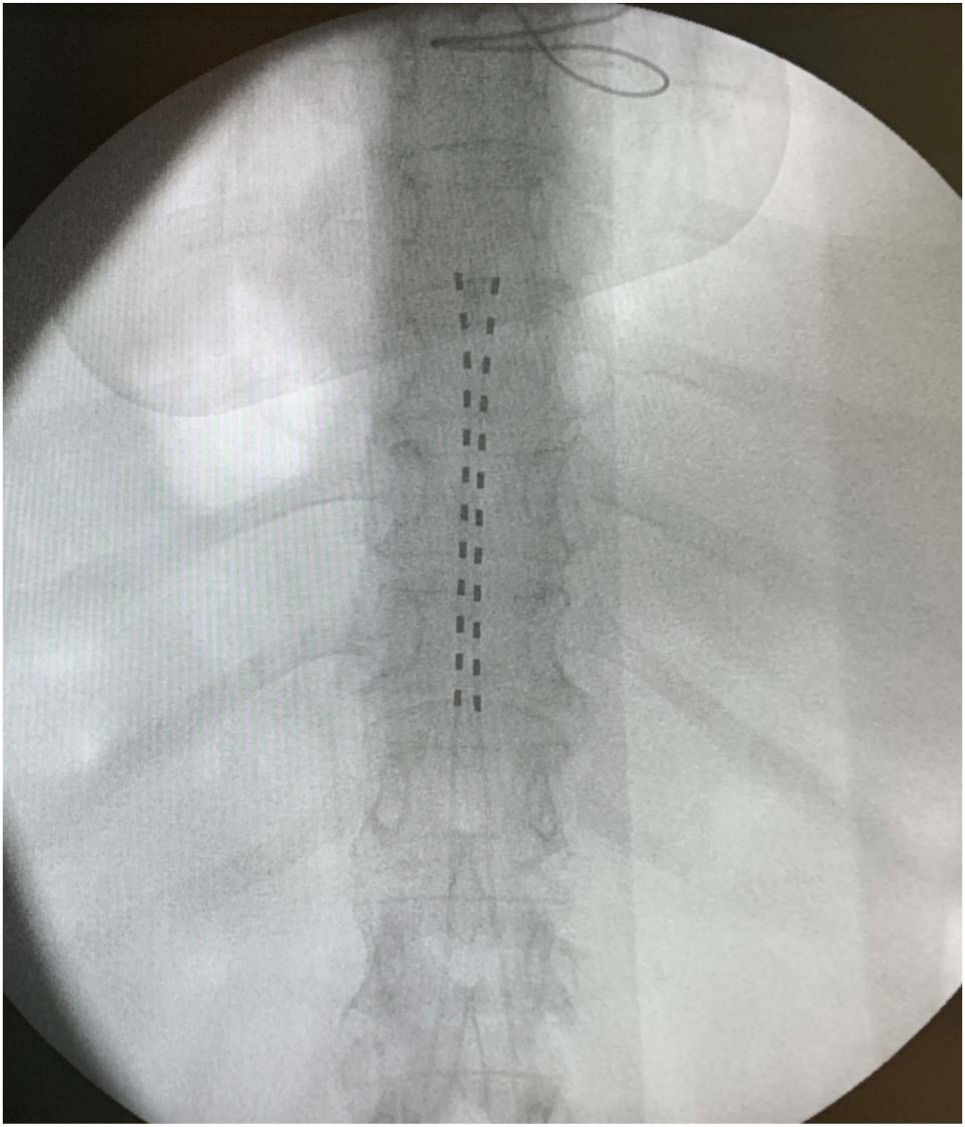
Anteroposterior radiographic view of the implanted closed-loop SCS in the patient, consisting of two 12-pole electrodes, with the upper electrode positioned at the lower endplate of T8. SCS, spinal cord stimulation.

In the operating room, a multidisciplinary team composed of cardiologists, anesthesiologists specialized in pain management, and engineers from both the ICD and SCS system teams was present. Although a trial phase was considered, we opted for a single-step implantation procedure to reduce risk exposure and minimize adverse events. Two twelve-pole leads were inserted percutaneously and positioned along the anatomical midline at the lower vertebral plate of T8 (Fig. 1). A protective polarity on the cathode (+) (−) (+) was established at the proximal ends of the leads, closest to the heart. After confirming pain coverage through a simple interview with the patient, an SCS trial was conducted. SCS parameters were set to deliver maximum energy intensity: frequency was set to 30 Hz, pulse width progressively increased beyond 500 μS, and the amplitude of both leads was adjusted incrementally to a combined total of 29.4 mA. The ICD was originally programmed in the VVI mode with a lower pacing rate of 40 bpm and the following tachycardia therapy settings: A ventricular tachycardia zone above 185 bpm with 6 antitachycardia pacing sequences followed by up to 6 shocks, and a ventricular fibrillation zone above 230 bpm with 1 antitachycardia pacing attempt and up to 6 shocks. During the procedure, tachycardia therapies were deactivated to prevent inappropriate shocks, and ventricular sensitivity was reduced to the minimum programmable value of 0.4 mV. On completion of the SCS implantation procedure, all ICD parameters were restored to their original settings. Noise detection in the ICD was thoroughly monitored during SCS tests, and no undersensing of the myocardial potentials or oversensing of impulses from the continuously active neurostimulator at maximal output in unipolar mode was documented. Thus, no interference was observed between the 2 devices.

Three months after the implantation, the patient reported 80% relief of her pain, as well as an improvement in her quality of life. She had not experienced any ventricular arrhythmias or oversensing episodes. The SCS parameters were as follows: frequency 30 Hz, pulse width 320 μS, amplitude variations of 0.3 mA (maximum of 25 mA), and a maximum voltage of 15 V. Both the SCS and ICD systems were functioning effectively, with no interference or complications reported.

## 3. Discussion

The widespread increase in cardiovascular pathologies requiring implantable devices, combined with the chronic pain stemming from these conditions or other disabling diseases—both of which are expected to rise alongside increasing life expectancy—suggests that the combined use of cardiac devices and SCS will continue to grow in the coming years.^5,8^

Although interferences between cardiac devices and SCS have been reported, these are isolated cases when analyzed in a broader context. In 2021, Martens et al.^5^ conducted what is likely the most comprehensive review on this topic to date. Among the 22 selected articles, interference was observed in only 3 case reports. Wenchun Qu et al.^9^ reported a patient in whom SCS affected the ventricular sensing of an ICD, while Molon et al.^7^ observed damage to an SCS device after a series of ICD shocks triggered by episodes of supraventricular tachycardia. Similarly, Romanó et al.^11^ reported intermittent inhibition in a pacemaker after increasing the stimulation voltage of the SCS device to achieve pain relief. By contrast, no adverse events related to the efficacy or safety of the devices were detected in the remaining 34 case reports or the 2 included patient cohorts (comprising 27 and 22 patients, respectively).

Despite the favorable evidence regarding the combined use of SCS and cardiac devices, there seems to be persistent doubt among specialists about the safety of these strategies. It is possible that the concerning results of the aforementioned cases, and even those from other neurostimulation strategies, have been overgeneralized. Specifically, transcutaneous electrical nerve stimulation (TENS), which is also used for pain relief, seems contraindicated in patients with ICDs because of its misinterpretation of neurostimulation signals as tachycardia or extra beats, potentially leading to inappropriate shocks. Some authors hypothesize that the higher amplitudes of TENS, compared with those of SCS, may explain this phenomenon.^12^ Consequently, organizations such as the American Heart Association and SCS manufacturers warn of the potential risks associated with implantation in patients with ICDs.^12^ Understandably, these concerns may lead to distrust or reluctance among clinicians when making clinical decisions.

Regarding the choice of the SCS device, we opted for a closed-loop system because of its ability to adjust the neural dose and its higher efficacy in mitigating chronic pain compared with open-loop systems, as demonstrated in Mekhail et al.'s^6^ clinical trial conducted in 2019. Although it could be argued that an open-loop system might favor patient safety by avoiding variations in the delivered current, the readjustments of the current did not pose a higher risk of interference with the ICD, as the maximum intensities were tested in the operating room without any incidents between the 2 devices. Given the potentially severe complications that interference between these devices could cause, and the fact that there are no case reports involving closed-loop systems, we believe it was our duty to report this case testing this branch of SCS devices.

In this case report, we have demonstrated firsthand that, by taking certain precautions, the combined use of a closed-loop SCS device and an ICD can be safe and ensure effective treatment. Our recommendations include: (1) Configuring both devices in the bipolar mode and choosing an SCS device that can be placed in the lumbar region to minimize risks.^5^ (2) Performing rigorous intraoperative testing at the SCS device's maximum energy intensity to ensure that both devices function correctly and verifying whether SCS artifacts could mask any arrhythmia, thereby preventing inappropriate ICD therapies. However, we personally discourage defibrillation because of its risks, which outweigh the potential benefits.^2,3^ (3) Quarterly monitoring to confirm that the SCS device functions correctly after potential ICD shocks and to ensure that ICD functionality is unaffected by changes in SCS parameters to maintain pain relief. Patients should also be instructed to promptly report any deviations from their baseline perception of the functioning of either device, no matter how minor.

In conclusion, the pain management strategy using a closed-loop SCS in patients with an ICD was successfully implemented with no interference between the 2 devices.

## Disclosures

The authors have no conflict of interest to declare.
